# Hard-on-Hard Lubrication in the Artificial Hip under Dynamic Loading Conditions

**DOI:** 10.1371/journal.pone.0071622

**Published:** 2013-08-07

**Authors:** Robert Sonntag, Jörn Reinders, Johannes S. Rieger, Daniel W. W. Heitzmann, J. Philippe Kretzer

**Affiliations:** 1 Laboratory of Biomechanics and Implant Research, Clinic for Orthopedics and Trauma Surgery, Heidelberg University Hospital, Heidelberg, Germany; 2 Heidelberg Motion Lab, Clinic for Orthopedics and Trauma Surgery, Heidelberg University Hospital, Heidelberg, Germany; Glasgow University, United Kingdom

## Abstract

The tribological performance of an artificial hip joint has a particularly strong influence on its success. The principle causes for failure are adverse short- and long-term reactions to wear debris and high frictional torque in the case of poor lubrication that may cause loosening of the implant. Therefore, using experimental and theoretical approaches models have been developed to evaluate lubrication under standardized conditions. A steady-state numerical model has been extended with dynamic experimental data for hard-on-hard bearings used in total hip replacements to verify the tribological relevance of the ISO 14242-1 gait cycle in comparison to experimental data from the *Orthoload* database and instrumented gait analysis for three additional loading conditions: normal walking, climbing stairs and descending stairs. Ceramic-on-ceramic bearing partners show superior lubrication potential compared to hard-on-hard bearings that work with at least one articulating metal component. Lubrication regimes during the investigated activities are shown to strongly depend on the kinematics and loading conditions. The outcome from the ISO gait is not fully confirmed by the normal walking data and more challenging conditions show evidence of inferior lubrication. These findings may help to explain the differences between the *in vitro* predictions using the ISO gait cycle and the clinical outcome of some hard-on-hard bearings, e.g., using metal-on-metal.

## Introduction

**Figure 1:**
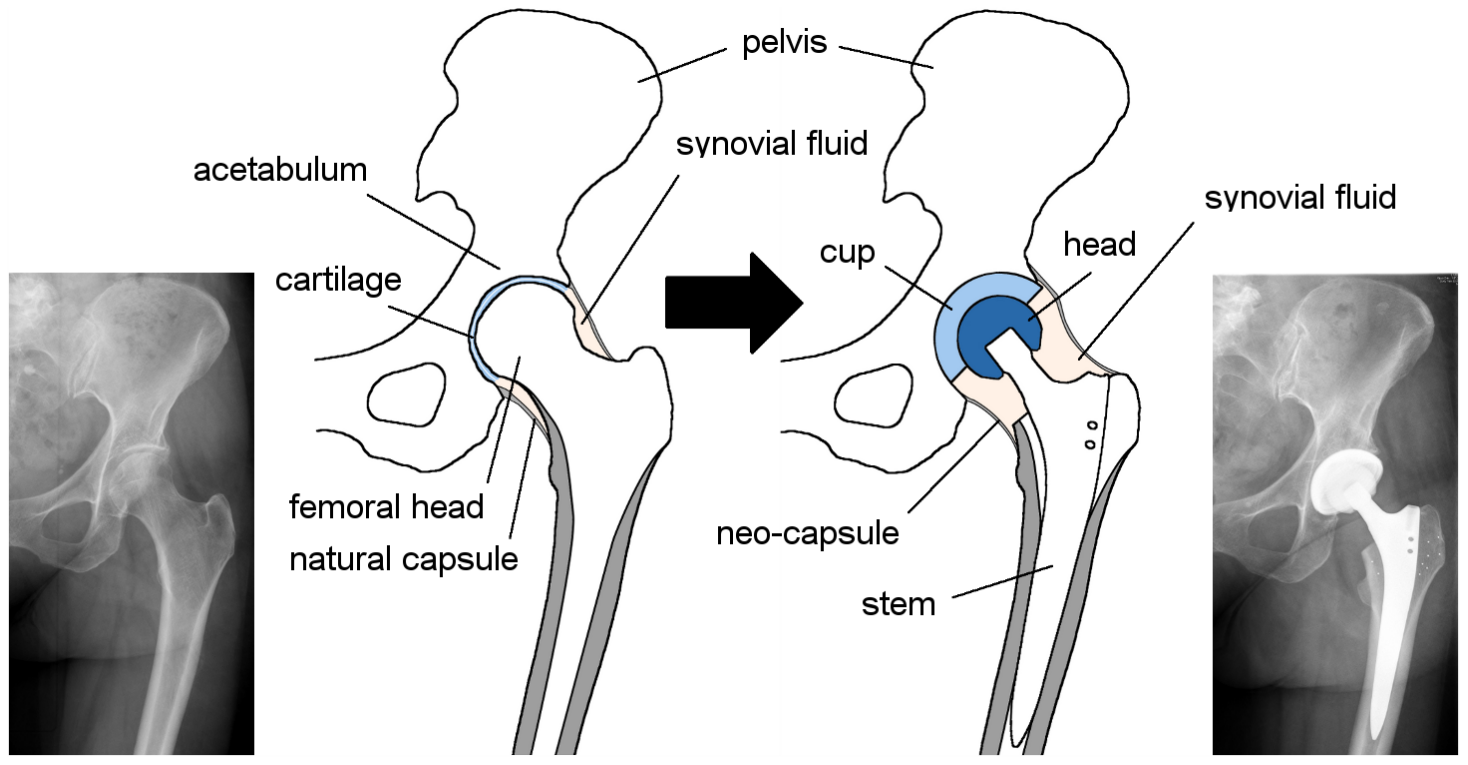
Hip articulation system; natural joint (left); total hip replacement (THR) (right).

The hip joint is probably the best studied joint of the human body. Unlike the knee where the kinematics are highly driven by the soft tissues, it naturally represents a ball-in-socket configuration with three rotational degrees of freedom. Gait analysis and experimental studies have provided profound insights into the hip’s kinematics as well as the joint forces under various loading conditions, such as normal walking or climbing stairs [1], [2]. Obviously, the bio-tribological system is considerably changed after implantation of a total hip replacement (THR) (Figure 1). This is likely to influence the clinical outcome as the compressive and permeable natural cartilage is replaced by a harder bearing couple made of a combination of ultra-high molecular weight polyethylene (UHMWPE), CoCr alloys or ceramics of various head diameters. In recent years, the osteolysis potential of conventional UHMWPE bearings [3] has led to the promotion of alternatives: besides cross-linking of UHMWPE, so-called hard-on-hard bearings made only of metal or ceramic components and the development of coatings and surface modifications are part of intense research activities [4]. However, in contrast to the convincing predictions from *in vitro* simulator wear testing [5], [6], the clinical outcome of some hard-on-hard bearings varies and is to some extent surprising [7], [8]. Friction and wear are therefore not only influenced by the external loads and kinematics applied to the joint, but also by the materials’ properties and the ability of the system to build up a load-bearing lubrication film which separates both bearing surfaces. The thickness of the lubrication film as well as the surface roughness determine whether direct contact occurs (boundary lubrication), if a fluid film is built up without fully separating the surfaces (mixed lubrication), or, in the case of a complete separation by a load bearing film (fluid film lubrication) where friction and wear are reduced to a minimum.

An unintentional increase in friction occurs if the lubrication film breaks down and both bearing partners come into contact with each other. In that case, high torsional loads are transmitted to the interface between implant and bone or to the taper at the head-stem interface, as recently reported for large diameter metal-on-metal bearings [9]. Malpositioning of the implant is one reason for poor lubrication and may lead to microseparation of the joint and an accelerated mechanical loosening especially in combination with insufficient bone quality. In addition, friction resulting from poor lubrication conditions is likely to be related to an increase in wear which is known to have a direct impact on the clinical performance of a joint [10], [3].

Measurement of the film thickness *in vitro* has been reported in several studies in which there were some limitations compared to the physiological situation. Most of these studies used pendulum simulators which subject the bearing to one single rotation combined with a static or dynamic axial load [11], [12]. From the friction values obtained in their experiments, the authors inferred to the mode of lubrication within the hip joint. Other attempts aim at directly measuring the gap between both bearing surfaces by optical [13] or conductive methods [14]. However, these approaches display significant limitations that directly influence the tribo-system, such as the modification of the bearing surfaces for physical measurements or use of a simplified load model that does not fully consider the *in vivo* conditions. So far, experimental lubrication studies mainly serve to provide a qualitative comparison of different bearing materials regarding their potential to build up a lubrication film but do not seem to be conclusive enough to represent absolute models.

Numerical methods have also been developed. Among them are complex approaches using computational fluid dynamics (CFD) which are based on the standard Reynolds equation, describing the pressure distribution in a fluid film, and an elastic deformation in order to derive detailed material aspects, e.g. by the finite element method (FEM). They have been developed for different independent conditions and analyses, e.g. fluid film lubrication, mixed lubrication or wear analysis [15]–[18]. However, an overall numerical model combining the use of the different approaches does not exist [19]. Thus, complex numerical analyses are currently very time-consuming and are as of yet still quite limited.

On the other hand, simplified theoretical approaches offer a good option to evaluate bio-tribological parameters, such as implant size or load conditions. They are mainly based on the early work of Hamrock and Dowson from the late 1970s who also created the term of *bio-tribology* meaning ‘all aspects of tribology related to biological systems’ [20]. They were the first to develop the elasto-hydrodynamic lubrication (EHL) theory by the simultaneous solution of the elasticity and the generalized Reynolds equations. Within this study, the approach has been applied to determine the lubrication regimes of different artificial joints [21]. The steady-state numerical model is used and extended by a dynamic loading aspect. The study aims to compare the lubrication regimes during dynamic ISO gait with those of more realistic loading data from patient measurements during normal walking, climbing stairs and descending stairs. It is hypothesized that the gait cycle as defined by the ISO produces lubrication conditions that do not fully represent physiological lubrication and may thus be part of the inability to predict clinical function of modern hard-on-hard bearings.

## Materials and Methods

A steady-state numerical model for calculation of the mode of lubrication [22] was extended by a dynamic loading and velocity profile. The lubrication regimes were approximated for all investigated loading conditions using the ratio of the minimal lubrication film thickness and the components’ arithmetic mean roughness R_a_ (*λ-ratio*), a parameter representing relative film thickness [19].
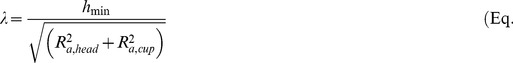
(2.7)


**Figure 2:**
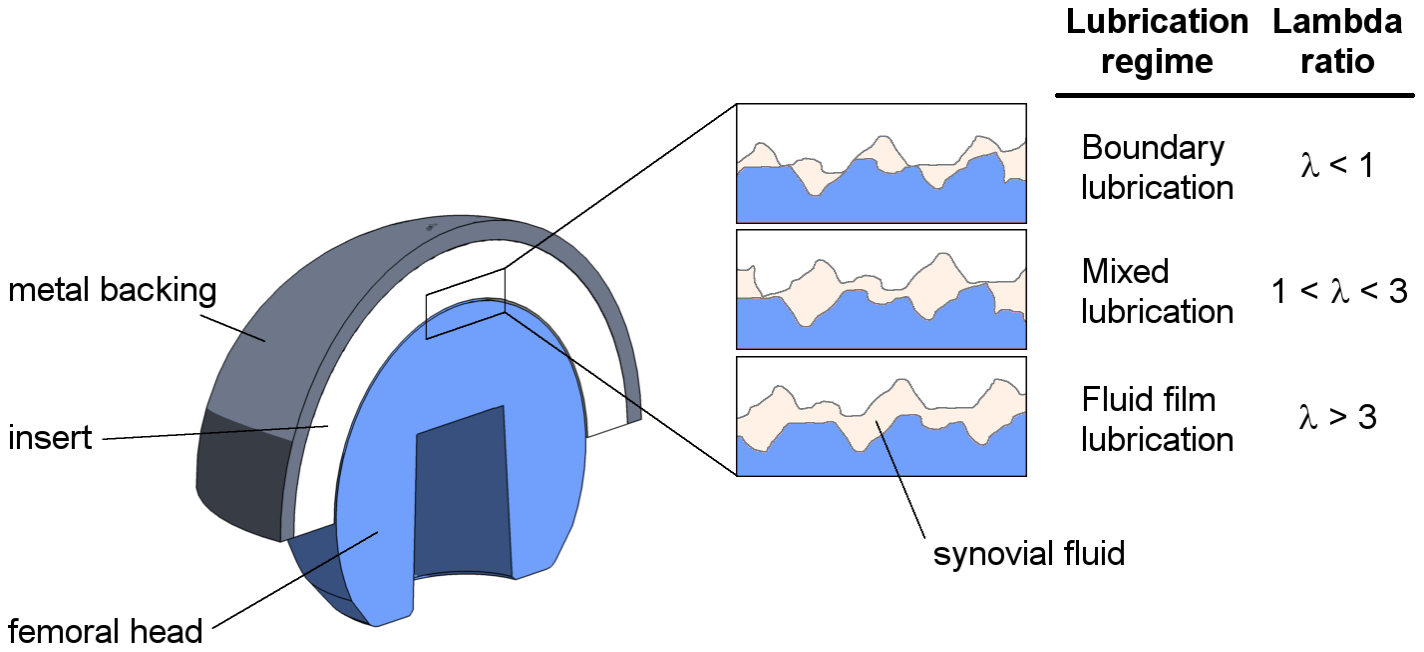
Correlation lambda ratio to lubrication regime [18].

Based on the *λ*-ratios, the lubrication regime was estimated according to Figure 2.

Minimal lubrication film thickness was calculated using the Hamrock-Dowson formula for iso-viscous and elastic lubrication for a whole gait cycle contingent upon the variation in the entraining velocity *u* and the applied axial load *L*, where *η* is the viscosity of the lubricant (here: 0.003 Pa s) [20], [22].
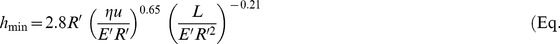
(2.6)


**Figure 3:**
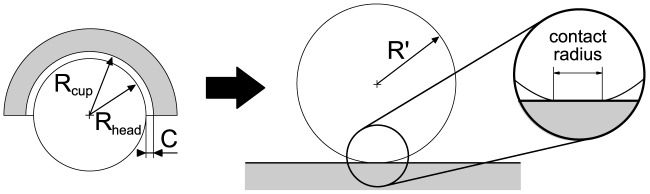
Head-cup configuration (left); ball-on-plane model (right).



Even when taking standard activities into account, such as normal walking, unexpected high contact pressures are generated [23]. They produce relevant local deformations that should not be disregarded in a theoretical approach, even for hard-on-hard bearings. Therefore, a ball-on-plane model (Figure 3) was used with an equivalent radius *R’* as well as an equivalent Young’s modulus *E’* which are defined as follows [21]:

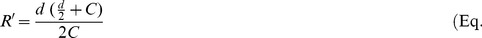
(2.2)

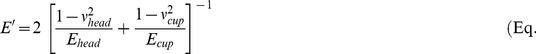
(2.3)


Heads and cups of an established hip implant system (Pinnacle, DePuy, Warsaw, IN, USA) with a nominal diameter of 36 mm were measured in a high-precision coordinate measuring machine (MarVision MS222, Mahr, Göttingen, Germany). Four implant pairs of each hard-on-hard bearing type were investigated: metal-on-metal (MoM), ceramic-on-metal (CoM) and ceramic-on-ceramic (CoC). Relevant material properties of the metal (CoCr) and ceramic (Biolox Delta) that are used for the numerical model are listed in Table 1. Radial clearance *C* was calculated as the difference between the cup (*R_cup_*) and head radii (*R_head_*) for the nominal diameter *d* of 36 mm.
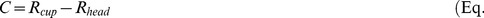
(2.1)


**Table 1:** Material parameters of the investigated metal and ceramic materials.

	Young’s modulus E	Poisson’s ratio ν	Surface roughness R_a_
CoCr	230 GPa	0.3	0.012 µm
Biolox Delta	358 GPa	0.2	0.008 µm

Four different load profiles were investigated:

1. ISO gait (from standard THR wear testing according to ISO 14242-1:2012)
2. Normal walking
3. Climbing stairs
4. Descending stairs

For the cases b., c. and d., the load profiles were taken from the *Orthoload* online database (www.orthoload.com) which provides force and moment data from multiple THR patients that received instrumented hip prostheses. Mean values for each loading condition were taken from the available patient data (Table 2). Kinematical data of stair ascent and descent was determined with the help of conventional instrumented gait analysis (CGA) at the Heidelberg Motion Lab. At least 8 stair ascents and descents at self-selected speed of the subjects were monitored on a custom-made 80 cm-wide staircase consisting of 5 steps of 15 cm height and a step distance of 32 cm. A 12 camera system (Vicon, Oxford Metrics, UK) operating at 120 Hz was used for 3D motion capture. Marker trajectories were processed using the conventional software Plugin-Gait (PiG, Vicon, Oxford Metrics, UK) following Kadaba et al. to obtain joint kinematics of the hip [24]. All subjects (14 male/7 female; mean height 173.5 cm±8.3 cm; mean weight 73.6 kg±12.4l g; mean age 30.5y±9.2y) were provided with the same type of shoes (Deichmann, Essen, Germany) during measurements. Both sides were analyzed to extract the relevant data: extension/flexion, abduction/adduction and internal/external rotation angles.

**Table 2:** Patient data from *Orthoload* online database and gait analysis.

	Load data	Kinematics
Source	www.orthoload.com	Gait analysis
**Normal walking**		
No. of patients	7	14
Sex	2f + 5m	3f + 11m
Weight	510-1010 N	516-833 N
**Walking upstairs**		
No. of patients	7	15
Sex	1f + 6m	6f + 9m
Weight	650-1010 N	516-1074 N
**Walking Downstairs**		
No. of patients	7	16
Sex	1f + 6m	7f + 9m
Weight	650-1010 N	516-1074 N

The wear track of an observed point on the head’s surface, such as the main contact point, was calculated as a function of hip angles of the four gait patterns and the velocity *u* of that point was determined numerically under consideration of the known mean stride time over the whole gait cycle (Mathworks MATLAB R2009b, Natick, MA, USA). The following rotation matrix *M* was used:



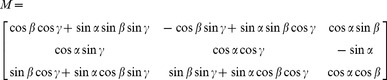
(2.5)


where α represents the flexion-extension, β the abduction-adduction and γ the internal-external rotation angle.

These results were used in the following as boundary conditions for the numerical model.

The fluid’s viscosity is known to be thixotropic, i.e. decreasing viscosity with increasing shear stresses in the fluid [25]. Using the present approach, the synovial fluid was considered to be iso-viscous and Newtonian as changes in fluid pressure within the expected range are assumed not to have a relevant impact on the lubrication analysis [22].

## Results

**Figure 4:**
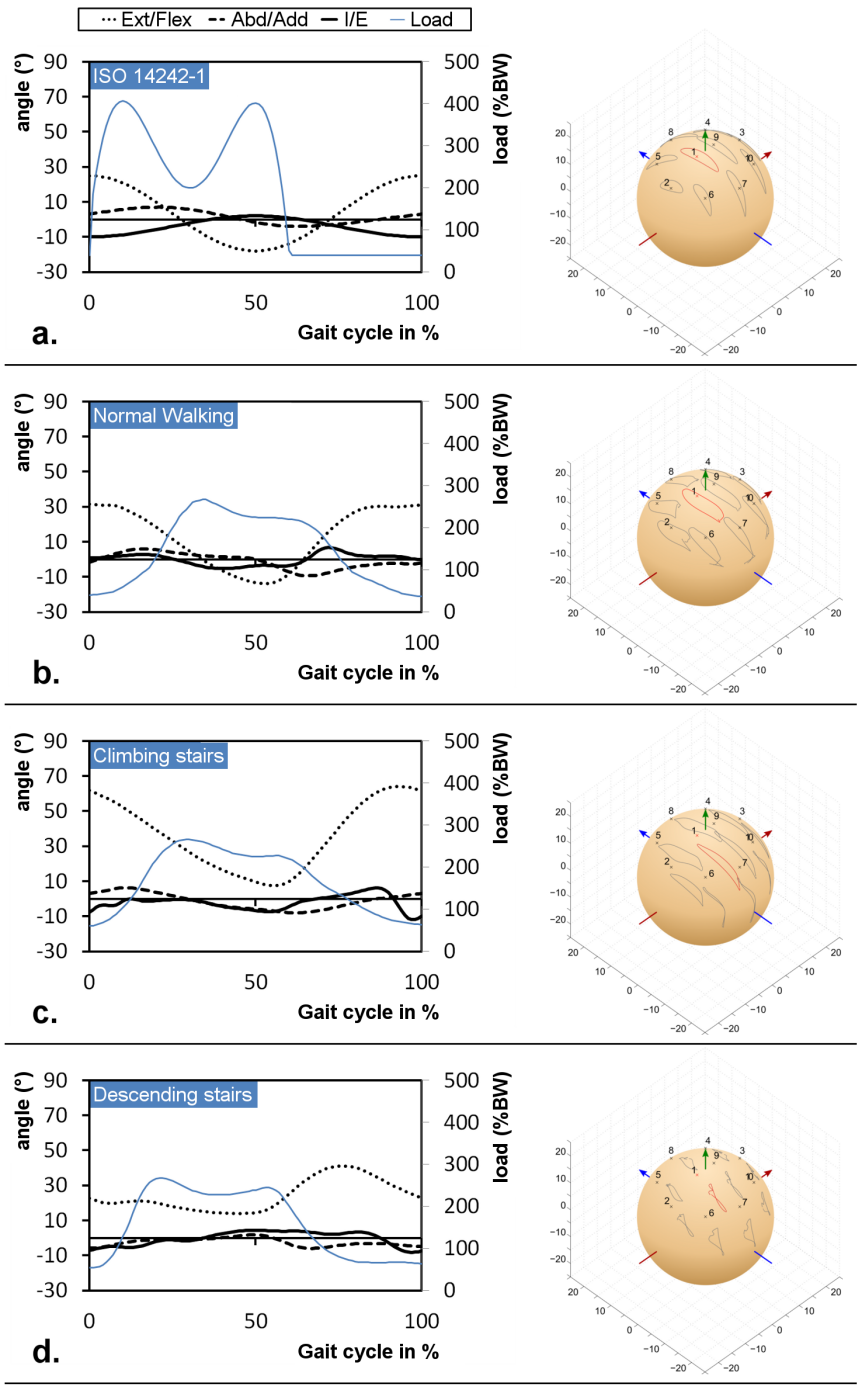
Kinematics and contact path for a. ISO gait, b. normal walking, c. climbing stairs and d. descending stairs.

**Figure 5:**
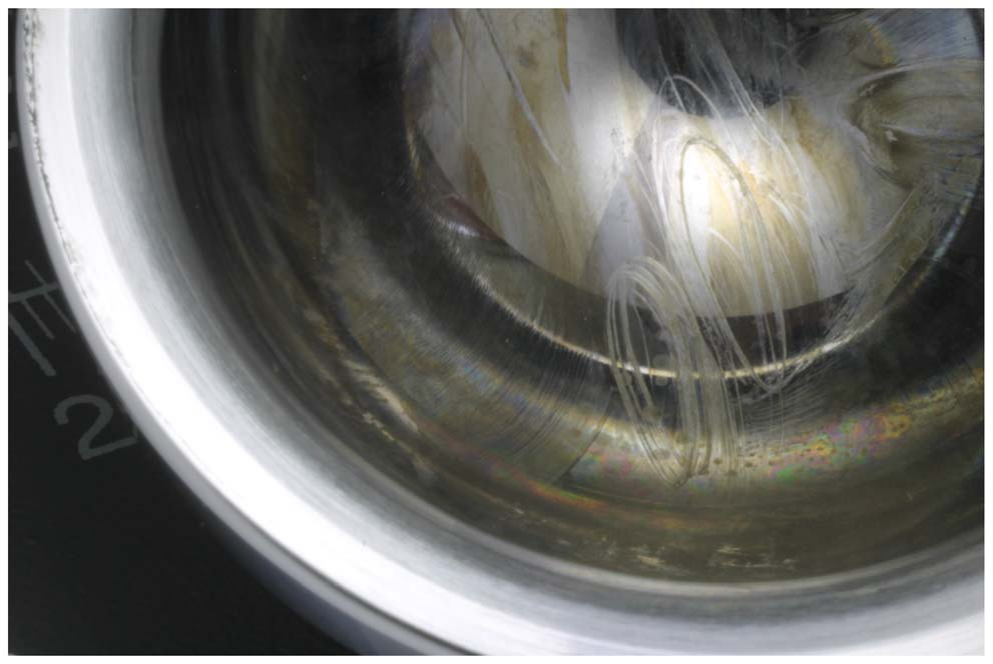
Elliptical contact paths on a metal-on-metal bearing after *in vitro* simulator testing.

Radial clearances from CMM measurements of the investigated 36 mm head-cup configuration varied from 37.8 µm±1.0 µm for MoM, 51.1 µm±2.0 µm for CoM and 23.8 µm±3.1 µm for CoC. The kinematics data for each activity during one gait cycle showed large variation between the load profiles with a 1.5 fold increase of the ISO peak load compared to the mean patient measurements during normal walking (Figure 4). However, the contact paths of various points, including the main contact point (#1), seem to be quite consistent regarding the ISO and corresponding normal walking while the other activities differed as expected. These elliptical paths were also observed after *in vitro* testing under water lubrication using the ISO profile in a hip simulator (Figure 5).

**Figure 6:**
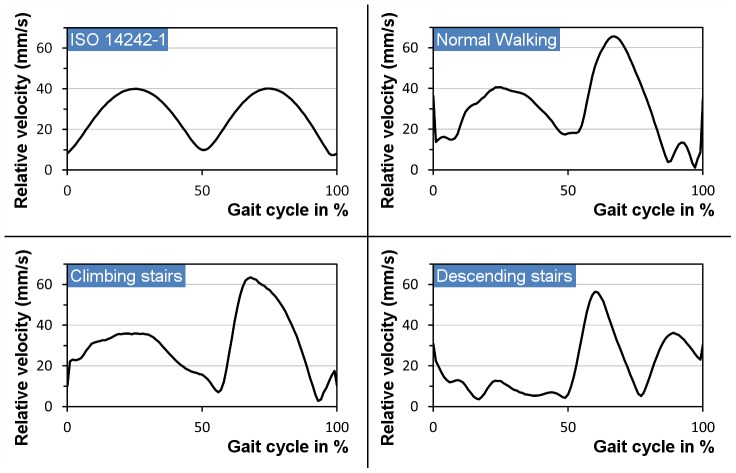
Relative velocities between the main contact point on the head and the insert.

The velocities of the main contact point that are used as the boundary condition for the calculation of the lambda ratio clearly illustrate the kinematical differences after patient measurements compared to the ISO kinematics (Figure 6).

**Figure 7:**
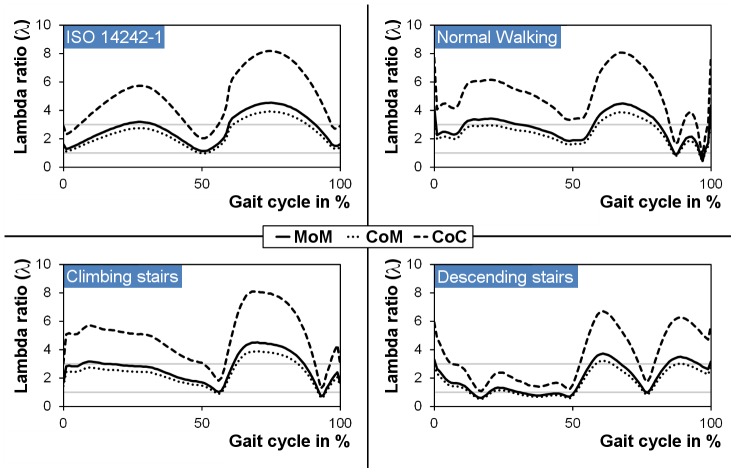
Lambda-ratio of different hard-on-hard bearings for dynamic loading conditions.

For a full gait cycle according to ISO 14242-1, the minimal fluid-film thickness was calculated to be between 19–77 nm in the case of a MoM, 14–56 nm for CoM and 23–92 nm for a CoC bearing. As could be expected, the fluid film thickness was generally higher during the swing phase as compared to the stance phase due to low external loads at increased velocities. All combinations therefore showed fluid film lubrication at least during the swing phase (Figure 7). The lubrication results from the ISO conditions were mainly confirmed by the experimental data (normal walking profile) from the *Orthoload* online database and the gait laboratory, even though the load profiles are different. However, at the end of the normal walking stance phase (87–97%), the fluid film lubrication breaks down as the velocity tends to zero. The data for climbing and descending stairs clearly differentiates these activities from the conditions of normal walking as the angular rotations differ to a significant extent (Figure 4). These effects are especially observed for the profile of descending stairs where lubrication is poor during the entire stance phase (Figure 7).

## Discussion

**Figure 8:**
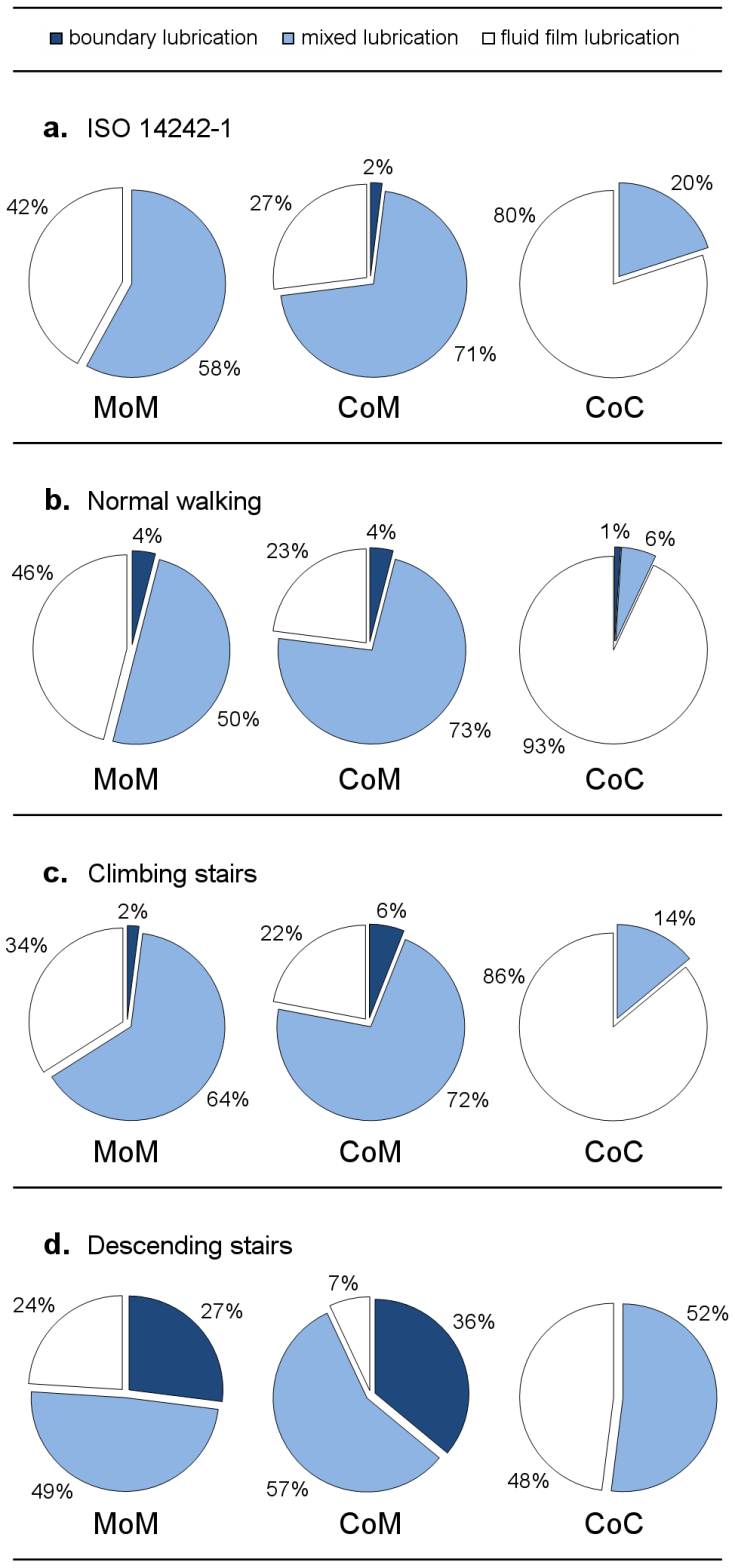
Distribution of lubrication regimes for hard-on-hard bearings relative to the full gait cycle.

The ISO kinematics represents a standardized condition for various applications, such as wear testing, working in the mixed or fluid film lubrication regime over the whole gait cycle and shows, as expected, an even and regular pattern of the lambda ratio. This was also confirmed over a wide range by the experimental patient measurements during normal walking conditions where, however, short periods of solid contact (boundary lubrication) exist which may be the cause for increased frictional stress at the interfaces to the bone and between modular components. This effect is further supported in the case of other investigated activities such as climbing stairs or descending stairs with up to 36% of the gait cycle working under boundary lubrication conditions, i.e. solid-solid contact (Figure 8). The present findings may help to explain why the clinical outcome of some modern hard-on-hard bearings differs that much from the theoretical or *in vitro* predictions which are reported under standardized ISO conditions [26]. In this context, the standard loading scenario of the ISO gait cycle does not cover any adverse conditions that simply appear in the daily life of a THR patient, e.g. start-stop conditions or microseparation. The data clearly shows that fluid film lubrication is inhibited during more challenging activities. This may be less relevant in the case of a polyethylene inlay against a CoCr or ceramic head which is anyway meant to work mainly under boundary or mixed lubrication [27]. Hard-on-hard bearings, however, develop their superior wear performance under conditions that favor the development of a load-bearing fluid film [28], [29]. Thus, wear testing of these bearings under idealized ISO conditions may not be representative and sufficient in the prediction of the clinical performance which may be better described using a kinematics protocol that favors partly boundary lubrication conditions.

Based on the theoretical data, relevant differences in the mode of lubrication are obvious for the studied hard-on-hard bearings. For all activities, the investigated CoC bearings seem to be advantageous which was confirmed theoretically and by experimental data [16], [29], [30], whereas the CoM bearings showed worse lubrication conditions even compared to MoM. All of the bearings work at least for 20% of the gait cycle under fluid film lubrication, except the CoM while descending stairs. Under this condition, the overall lubrication is worst for all material combinations. As a result, when considering larger CoM as a potential bearing material, the frictional torque may be in the same range as reported or even higher as this is the case for large diameter MoM bearings [9].

The present results are calculated for the investigated standard hard-on-hard bearings and the theoretical model still contains some limitations: the deviation of roundness and surface wettability of the materials are not considered. Regarding lubrication of artificial joints, the viscosity of the synovial fluid plays a decisive role in the joint’s performance. It works very well in a cartilage-cartilage articulation and has displayed a non-Newtonian and thixotropic behavior under external loads, dependent on the local grade of joint degeneration and whether the joint has been replaced [31]. However, the synovial fluid is approximated as being iso-viscous and Newtonian [22]. In addition, the particular influence of proteins and hyaluronic acid composition seems to be important in the development of an extremely low coefficient of friction during a gait cycle [32]. Besides the basic fluid-mechanics, complex tribo-chemical mechanisms and interactions between the fluid and the bearing surfaces, such as adhesion of an organic tribo-layer, are discussed in the literature [33]–[35]. Material changes due to wear, e.g. modification of surface roughness over time, are not considered. Moreover, the investigated load profiles of THA patients from the *Orthoload* database were matched with a number of asymptomatic normal subjects from a different cohort that was measured in a gait laboratory. Even though different function levels regarding joint kinematics are a subject of controversy [36], [37], the individual profiles are consistent with each other within one cohort which is why the database used for this model is considered to be representative.

## Conclusion

In summary, CoC bearings showed the best bio-tribological performance when compared to MoM and CoM bearings of the same size and design according to the theoretical evaluation used in this study. However, none of the investigated bearings was able to fully work under fluid film lubrication during the entire gait cycle for the activities ‘ISO gait’, ‘normal walking’, ‘walking upstairs’ and ‘walking downstairs’. This means that solid contact is partly taking place in all bearing combinations. However, it is assumed that the divergence of the ISO and *in vivo* lubrication accounts for the insufficient performance to predict the clinical outcome of modern hard-on-hard bearings. More challenging activities favor periods of boundary lubrication and are considered more appropriate for a realistic pre-clinical testing scenario. The study provides a possible explanation as to why the performance of modern non-polyethylene bearings is hard to predict based on the ISO testing protocol. The frictional behavior may be one limiting factor, which needs to be confirmed by more complex experimental data.
